# Loss of FOXF1 expression promotes human lung-resident mesenchymal stromal cell migration via ATX/LPA/LPA1 signaling axis

**DOI:** 10.1038/s41598-020-77601-1

**Published:** 2020-12-04

**Authors:** Pengxiu Cao, Natalie M. Walker, Russell R. Braeuer, Serina Mazzoni-Putman, Yoshiro Aoki, Keizo Misumi, David S. Wheeler, Ragini Vittal, Vibha N. Lama

**Affiliations:** Division of Pulmonary and Critical Care Medicine, Department of Internal Medicine, University of Michigan Health System, 1500 W Medical Center Drive, 3916 Taubman Center, Ann Arbor, MI 48109-0360 USA

**Keywords:** Cell biology, Molecular biology, Medical research

## Abstract

Forkhead box F1 (FOXF1) is a lung embryonic mesenchyme-associated transcription factor that demonstrates persistent expression into adulthood in mesenchymal stromal cells. However, its biologic function in human adult lung-resident mesenchymal stromal cells (LR-MSCs) remain to be elucidated. Here, we demonstrate that FOXF1 expression acts as a restraint on the migratory function of LR-MSCs via its role as a novel transcriptional repressor of autocrine motility-stimulating factor Autotaxin (ATX). Fibrotic human LR-MSCs demonstrated lower expression of FOXF1 mRNA and protein, compared to non-fibrotic controls. *RNAi-*mediated *FOXF1* silencing in LR-MSCs was associated with upregulation of key genes regulating proliferation, migration, and inflammatory responses and significantly higher migration were confirmed in *FOXF1*-silenced LR-MSCs by Boyden chamber. ATX is a secreted lysophospholipase D largely responsible for extracellular lysophosphatidic acid (LPA) production, and was among the top ten upregulated genes upon Affymetrix analysis. *FOXF1*-silenced LR-MSCs demonstrated increased ATX activity, while *mFoxf1* overexpression diminished ATX expression and activity. The *FOXF1* silencing-induced increase in LR-MSC migration was abrogated by genetic and pharmacologic targeting of ATX and LPA1 receptor. Chromatin immunoprecipitation analyses identified three putative *FOXF1* binding sites in the 1.5 kb *ATX* promoter which demonstrated transcriptional repression of ATX expression. Together these findings identify FOXF1 as a novel transcriptional repressor of ATX and demonstrate that loss of FOXF1 promotes LR-MSC migration via the ATX/LPA/LPA1 signaling axis.

## Introduction

Mesenchymal cells are an important component of cellular niches in adult organs and are being increasingly recognized to display tissue-specific transcriptome and functions. We have previously demonstrated that human adult lung contains a population of resident, long-lived mesenchymal stromal cells (MSC) with clonal multi-lineage differentiation potential^1^. MSCs derived from adult lungs exhibit unique mesenchymal transcriptional signature suggesting their lung-specificity and origin from embryonic lung mesenchyme^1,2^. Expansion and mobilization of lung-resident mesenchymal stem cells (LR-MSCs) is noted during conditions of lung injury and fibrosis^2,3^, and the lipid mediator lysophosphatidic acid (LPA) has been identified as a key inducer of LR-MSC migration^4^. LR-MSCs can regulate LPA expression in an autocrine manner by secretion of Autotaxin (ATX), a lysophospholipase D that enzymatically produces LPA from extracellular lysophosphatidylcholine^5^. We have recently demonstrated that ATX expression is upregulated in LR-MSCs derived from diseased lungs and can drive β-catenin activation via downstream LPA/LPA1 signaling^5^. While these data shed light on mechanisms of MSC mobilization and activation, transcriptional regulation of MSCs under homeostatic conditions remain to be identified.

The Forkhead Box (Fox) family of transcription factors is a group of proteins that share a common DNA binding domain termed a winged-helix or forkhead domain, with FOXF1 being a mesenchyme-specific, putative transcription factor^6,7^, which plays a critical role in lung development. In mice, FOXF1 expression is noted in the lateral mesenchyme at embryonic day 9.5 and its deficiency is associated with defects in branching morphogenesis of the lung^8,9^. *FOXF1* is unique among the embryonic lung mesenchyme-associated transcription factors in that it demonstrates persistent expression in the mesenchymal cells of an adult lung, and we have reported that LR-MSCs derived from human adult lungs express ~ 35,000-fold higher *FOXF1* mRNA than bone-marrow MSCs^5^. However, the significance of FOXF1 expression in adult human LR-MSCs remains to be elucidated.

In this study, we investigated the mechanistic role of FOXF1 in regulating the biologic functions of lung-resident mesenchymal stem cells. Our investigations identify FOXF1 as a transcriptional repressor, with loss of *FOXF1* promoting an activated mesenchymal phenotype accompanied by intense mitogenic activity, higher rates of cellular migration, proliferation, and the secretion of pro-inflammatory chemokines and cytokines. Importantly, our data demonstrates that *FOXF1* regulates the migration of LR-MSCs via its novel role as a transcriptional repressor of *ATX*.

## Materials and methods

### Human subjects and ethics statement

Informed consent was obtained directly from all human subjects after a full explanation of the study objectives and procedures. The study was carried out in accordance with relevant guidelines and regulations using a protocol for human studies approved by the University of Michigan Institutional Review Board (approval number HUM00042443) and was in compliance with the Helsinki Declaration.

### Isolation and culture conditions of LR-MSCs

LR-MSCs were isolated and characterized as previously described from bronchoalveolar lavage derived from lung transplant recipients with or without chronic lung allograft rejection^1–5,10,11^. LR-MSCs cultured in high-glucose DMEM (11965-118, Gibco) supplemented with 10% FBS, 100 U/ml penicillin/streptomycin, and 0.5% fungizone were utilized at passages 3–6. All methods were carried out in accordance with relevant guidelines and regulations.

### RNA interference

At 60–70% confluence, LR-MSCs were transfected with 100 nM *FOXF1*-specific (Stealth RNAi HSS142031, Invitrogen) or scrambled control siRNA (sc-37007, Santa Cruz), using Oligofectamine (12252-011, Invitrogen) and Opti-MEM I reduced serum medium. For double gene silencing, LR-MSCs were transfected with *FOXF1-*specific siRNA or scrambled control (100 nM each). 24 h later, LR-MSCs were transfected again with 100 nM of *ATX-*specific siRNA or scrambled control, incubated overnight, and then maintained in serum-free DMEM. Cells were subsequently assayed for migration rates after 48 h. RNA and protein lysates were harvested after 72 h for real-time PCR and immunoblotting analysis.

### Lentiviral transduction of LPAR1 short hairpin RNA

For lentivirus transduction, LR-MSCs were transfected with *FOXF1-*specific siRNA or scrambled control (100 nM each) and incubated for 48 h. Subsequently, the cells were infected with lentiviral vectors that contain either a control transduction particles-shRNA (Mission pLKO.1-puro, Sigma) or LPAR1-specific transduction particles-shRNA (Mission: Clone #: NM_057179, Sigma) in serum-free growth medium with 2.5 multiplicities of infection (MOI) using protamine sulphate as linker. After incubating for a period of 48 h, the cells were subsequently assayed for migration rates.

### Affymetrix analysis

Total RNA extracted from LR-MSCs transfected with *FOXF1*-specific or scrambled control siRNA were used to synthesize cDNA, followed by Affymetrix GeneChip expression profiling (U133 Plus 2.0 Array) at the University of Michigan DNA Sequencing Core. The affy and limma packages of bioconductor implemented in the R statistical environment were used^12–14^. The GO analysis was performed using conditional hypergeometric tests from the GO stats package of Bioconductor^15^. A cutoff of *p* values < 0.0001 was utilized to determine GO terms that were relevant. The microarray data has been  deposited in GEO under the Accession number GSE161903.

### RNA isolation and quantitative PCR

Total RNA was isolated from LR-MSCs using the RNeasy mini kit (74104, Qiagen) and cDNA synthesized utilizing the High Capacity cDNA reverse transcription kit (4368814, Applied Biosystems). Real-time PCR for *FOXF1*, *ATX* and *β-actin* were conducted with probes Hs00230962_m1, Hs00905125_m1, and 4310881E (Applied Biosystems), respectively in 1× Taqman gene expression master mix (4369016, Applied Biosystems). The *forward* and *reverse* primer sequences for the genes analyzed using SYBR Green PCR Master Mix (4,309,155, Applied Biosystems) are listed in Table 1. Relative mRNA expression for target genes was calculated as 2 log^− (Δ*C*t target mRNA − Δ*C*t β-actin)^.

**Table 1:** Primer sequences used in this study.

CCND1 forward	GCTGCGAAGTGGAAACCATC
CCND1 reverse	CCTCCTTCTGCACACATTTGAA
CCNB1 forward	AATAAGGCGAAGATCAACATGGC
CCNB1 reverse	TTTGTTACCAATGTCCCCAAGAG
CDK1 forward	GGATGTGCTTATGCAGGATTCC
CDK1 reverse	CATGTACTGACCAGGAGGGATAG
PEA15 forward	GGAGAGCCACAACAAGCTG
PEA15 reverse	CCATAGTGAGTAGGTCAGGACG
CCL5 forward	CCAGCAGTCGTCTTTGTCAC
CCL5 reverse	CTCTGGGTTGGCACACACTT
CCL7 forward	GAGAGCTACAGAAGGACCAC
CCL7 reverse	GTTTTCTTGTCCAGGTGCTTC
CCL8 forward	TGGAGAGCTACACAAGAATCACC
CCL8 reverse	TGGTCCAGATGCTTCATGGAA
CXCL10 forward	GTGGCATTCAAGGAGTACCTC
CXCL10 reverse	GCCTTCGATTCTGGATTCAG
CXCL11 forward	GACGCTGTCTTTGCATAGGC
CXCL11 reverse	GGATTTAGGCATCGTTGTCCTTT
PTGS2F forward	CAGGCAGAGATGATCTACCCTCCTC
PTGS2R reverse	GCAGCCAGATTGTGGCATACATC

### Immunoblotting and autotaxin activity assay

Whole cell lysates of LR-MSCs were extracted as reported previously^4^. Western blot was performed using primary antibodies against FOXF1 (AF4798, R&D, 1:1000), ATX (10005375, Cayman Chemical, 1:100), PCNA (2586, Cell Signaling Technologies, 1:1000), Cyclin D1 (sc-718, Santa Cruz Biotechnology, 1:1000), phospho-Histone H3 Ser10 (PA5-17869, Thermo Fisher, 1:500) and GAPDH (MAB374, Millipore, 1:5000). HRP-conjugated secondary antibodies used in this study included A5420 (anti-goat, Sigma, 1:5,000), A8924 (anti-mouse, Sigma, 1:20,000) and A0545 (anti-rabbit, Sigma, 1:10,000), respectively. ATX activity in the conditioned media was measured with a fluorogenic ATX substrate FS-3 as previously described^5^.

### Proliferation assay

LR-MSCs were transfected with *FOXF1* siRNA or scrambled control (100 nM each), and 24 h post-transfection, the mesenchymal cells were trypsinized and seeded at 5000 cells/well in 96 well plates. Cells were cultured in medium for 72 h and assayed with a CyQUANT NF Cell Proliferation Assay Kit (C35006, ThermoFisher Scientific) as per manufacturer´s instructions.

### Protein measurement in cell supernatant

Mesenchymal cells were transfected with *FOXF1*-specific or the scrambled siRNA in Opti-MEM I reduced serum medium. After overnight incubation, media was exchanged for serum-free DMEM for 48 h and the conditioned media was measured for CCL5 and CCL7 by ELISA according to the manufacturer’s protocols: R&D systems (Minneapolis, MN): Human CCL5/RANTES Quantikine ELISA Kit (DRN00B), Human CCL7/MCP-3 Quantikine ELISA Kit (DCC700). Absorbance was read with a SpectraMax M3 multi-mode microplate reader (Molecular Devices).

### Migration assay

Migration rates of LR-MSCs was measured in matrigel-coated transwells as previously described^4^. Briefly, transwells were freshly coated with matrigel overnight at 37 °C. LR-MSCs were transfected with *FOXF1*-specific or the scrambled siRNA for three days, trypsinized, and re-suspended in serum-free DMEM. 1 × 10^5^ cells were seeded into each upper chamber of the transwells, inserted into a 24-well plate containing DMEM supplemented with 5% FBS. Each condition was performed in triplicates. For migration assay involving PF-8380 treatment, LR-MSCs transfected with *FOXF1*-specific or scrambled siRNA were pre-treated with medium containing 1 µM PF-8380 (HY-13344, MedChem Express) for 30 min. They were then seeded into the transwell setup with 1 µM PF-8380 added to both the upper and lower chambers. 18 h later, LR-MSCs on the transwells were fixed and stained by Hema 3 staining kit (Fisher Scientific, Kalamazoo, MI). Finally, after the removal of the cells on the upper surface of the transwells using cotton swabs, the cells on the surface underneath were counted in five 200 × microscopic fields to quantify amount of cellular migration.

### Murine *Foxf1* overexpression

Mouse *Foxf1* was overexpressed in LR-MSCs by transfection of pShuttle A-CMV-*mFoxf1* utilizing Lipofectamine transfection reagent in Opti-MEM I reduced serum medium. The *mFoxf1* overexpression plasmids were a generous gift by Dr. Vladimir V. Kalinichenko, MD, PhD, Cincinnati Children’s Hospital Medical Center, OH. It should be noted that the human FOXF1 (NCBI: NP_001442, 379 amino acid) is 94% homologous to murine FOXF1a (NCBI: NP_034556.1, 353 amino acid)^16^. Dr. Kalinichenko’s lab analyzed human and mouse FOXF1 sequences and identified two highly conserved homologous regions containing potential transcription factor binding sites for the following families: basic leucine zipper CCAAT/enhancer binding protein β, winged helix/Forkhead Box A, zinc finger GATA, homeodomain Nkx2.5, and cut-homeodomain HNF-6 transcription factors^17^. Since Dr. Kalinichenko’s lab has well-documented evidence of the efficacy of this plasmid^16,18,19^, we have chosen to utilize this plasmid to overexpress FOXF1 in our LR-MSCs. Empty pShuttle A was used as control. Transfection efficacy was confirmed by immunoblotting techniques.

### Luciferase reporter assay

To examine the transcriptional functionality of 3 potential binding sites of *FOXF1* in ATX promoter, 1.5 kb ATX promoter plus 150 bp downstream of the transcriptional start site was cloned into pGAL4.23 vector to drive *luc2* luciferase gene expression. Three truncated versions of the ATX promoter (pATX1, pATX2 and pATX3) lacking one, two or all three potential *FOXF1* binding sites were generated to drive *luc2* luciferase gene expression in pGAL4.23. Plasmids were co-transfected with Renilla luciferase reporter pRL-TK (E224A, Promega) in LR-MSCs, and the luciferase activity was assayed 24 h post-transfection using Dual-Luciferase Reporter Assay System kit (E1960, Promega) by a Promega GloMax Explorer System Multimode Reader. The *luc2* luciferase activity was normalized to the control Renilla luciferase activity to represent the transcriptional activity of the promoters.

### Chromatin immunoprecipitation (ChIP) assay

ChIP assay was performed utilizing EZ-ChIP kit (17–371, Millipore) according to the manufacturer´s protocol. Briefly, LR-MSCs cultured in three 10 cm dishes at 80% confluence were cross-linked by 1% formaldehyde (10 min × RT), then quenched with 1 mM Glycine. Cells were pelleted and resuspended in SDS lysis buffer, and DNA sheared by sonication to reach ~ 200–1000 base pairs in length. Total fragmented DNA aliquots were incubated with anti-FOXF1 antibody (AF4798, R&D) or goat IgG (I5256, Sigma) as the background binding control at 4 °C overnight. Samples were subsequently incubated with Protein G agarose (4 °C × 1 h) followed by elution of protein/DNA complexes and heated at 65 °C × 5 h to reverse cross-linked complexes. Finally, DNA were purified and subjected to real-time PCR analysis using the following primers to detect binding at putative sites: CHIPSite1F, ACTAGATTCTAAGAATCTGTAATGAA; CHIPSite1R, GTAACAGAGTAGTGGCTCT; CHIPSite2F, AGAGCCACTACTCTGTTAC; CHIPSite2R, TTTTTGGCCTCTTCCTCAGCA; CHIPSite3F, ATGTGATACTAGGGACAGG; CHIPSite3R, AATGGCGTCAACCTCACCA.

### Statistical analysis

All data are presented as Means ± SEM. Statistical significance was assessed with Student’s paired two-tailed *t* test for comparing scrambled and *FOXF1*-silenced groups, or with one-way ANOVA and a post hoc Bonferroni test for 3 or more groups, unless specified otherwise using GraphPad Prism 8 software (La Jolla, CA). *p* < 0.05 was considered significant.

## Results

### FOXF1 as a transcriptional regulator of key functional pathways in LR-MSCs

**Figure 1:**
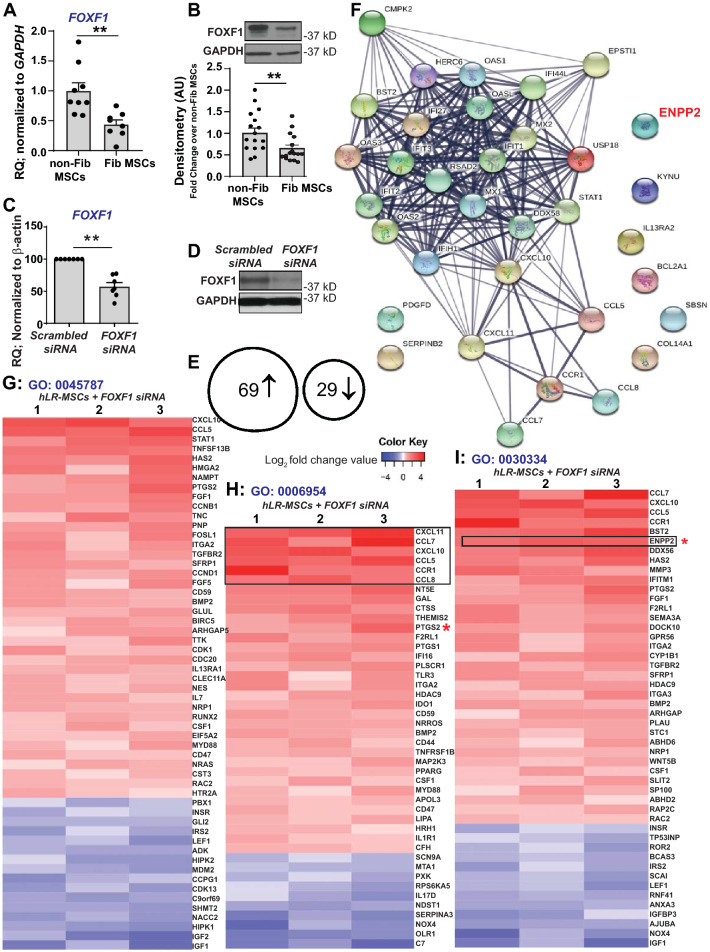
Loss of *FOXF1* promotes fundamental cellular processes in LR-MSCs. (**A**) mRNA was isolated from fibrotic and non-fibrotic LR**-**MSCs derived from bronchoalveolar lavage fluid of transplant patients, and *FOXF1* expression was analyzed by real-time PCR. Values: Means ± SEM; n = 9 (non-Fib-MSCs); n = 8 (Fib-MSCs); **p < 0.0034. (**B**) Protein lysates from fibrotic and non-fibrotic LR**-**MSCs were analyzed for FOXF1 and GAPDH by western blotting. Graph shows densitometry analyses of these immunoblots. Values: Means ± SEM; n = 16; **p < 0.0086. (**C**) LR**-**MSCs were transfected with scrambled or *FOXF1-*specific siRNA and confirmed by real-time PCR. Values: Means ± SEM; n = 7; ***p < 0.0005. (**D**) Protein lysates from (**A**) were subjected to immunoblotting with antibodies against FOXF1 and GAPDH. (**E**–**I**) Gene regulation due to *FOXF1* silencing was analyzed by Affymetrix gene array in 3 lines of LR-MSCs. Data reflects fold changes ≥ 1.5, and an adjusted *p* < 0.01. (**E**) Diagram showing the number of upregulated and downregulated genes. (**F**) Gene–gene interaction network (using STRING database) showing associations due to *FOXF1*-silencing. (**G**–**I**) Heatmaps showing two-fold Log changes are presented for positive regulation of cell cycle ((**G**) GO:0045787), inflammatory response ((**H**) GO:0006954), and regulation of cell migration ((**I**) GO:0030334). Note: Full length blots for Fig. 1B and Fig. 1D are provided in Supplementary Fig. S1 and S2.

We have previously demonstrated that lung mesenchyme associated embryonic transcription factor Foxf1 is uniquely expressed in human lung allograft-derived MSCs. Comparison of the MSCs derived from patients with and without chronic allograft rejection demonstrated lower expression of *FOXF1* transcripts (Fig. 1A) and protein (Fig. 1B) in fibrotic mesenchymal cells. In order to gain insight into the functional role of FOXF1 in human LR-MSCs, we used *RNAi-*mediated *FOXF1* silencing and Affymetrix whole transcriptome array analyses. LR-MSCs silenced for *FOXF1* demonstrated a 55% decrease in *FOXF1* mRNA expression (Fig. 1C) and a concomitant downregulation in FOXF1 protein (Fig. 1D). Affymetrix analyses of the RNA isolated from LR-MSCs transfected with *FOXF1*-specific or scrambled siRNA revealed that 98 probe sets had a fold change ≥ 1.5 and an adjusted p-value below 0.01. Of these probesets, 69 genes were upregulated and 29 genes were downregulated as a result of *FOXF1* silencing, thus suggesting that FOXF1 predominantly functions as a transcriptional repressor in LR-MSCs (Fig. 1E). We further filtered the dataset and focused on genes with a fold change above 2 or below − 2, and an adjusted p value below 0.05. This process revealed the top 35 differentially expressed genes, which were subsequently used to construct a gene–gene interaction network (STRING) to predict associations due to *FOXF1*-silencing (Fig. 1F). Gene ontology (GO) analysis was performed using conditional hypergeometric tests from the GO stats package of bioconductor^15,20^. Top ten GO terms in biological processes were widely modulated by *FOXF1* silencing including proliferation, inflammatory responses, and migration (Table 2). Proliferation/cell cycle GO analyses revealed predominant upregulation of genes involved in positive regulation of proliferation and the downregulation of genes implicated in cell cycle arrest (Fig. 1G). Loss of *FOXF1* in LR-MSCs induced an inflammatory response in these cells with upregulation of 34 of 44 genes associated with the inflammatory response GO term (GO:0006954) (Fig. 1H). A similar trend was noted with differentially expressed genes in positive regulation of cell proliferation and regulation of cell migration (GO:0008284 and GO:0030334, respectively) (Fig. 1I). Taken together, these data suggest a role for FOXF1 in fundamental cellular process of LR-MSCs including proliferation, cell cycle progression, inflammation, and migration.

**Table 2:** Gene ontology: biological processes significantly associated with *FOXF1* silencing in LR-MSCs.

Accession	GO term	Category size	Overlap	Odds ratio	p-value
GO:0030334	Regulation of cell migration	400	47	4.82	9.86E−04
GO:0044420	Extracellular matrix part	91	22	3.77	1.68E−06
GO:0071345	Cellular response to cytokine stimulus	334	60	2.6	2.47E−09
GO:0045787	Positive regulation of cell cycle	100	18	2.53	1.00E−03
GO:0006955	Immune response	851	131	2.27	8.51E−14
GO:0048638	Regulation of developmental growth	400	49	1.62	2.15E−03
GO:0006954	Inflammatory response	351	44	1.5	5.14E−05
GO:0008284	Positive regulation of cell proliferation	516	57	1.44	9.36E−03

### Loss of *FOXF1* induces migration and the expression and activity of ATX in human LR-MSCs

**Figure 2:**
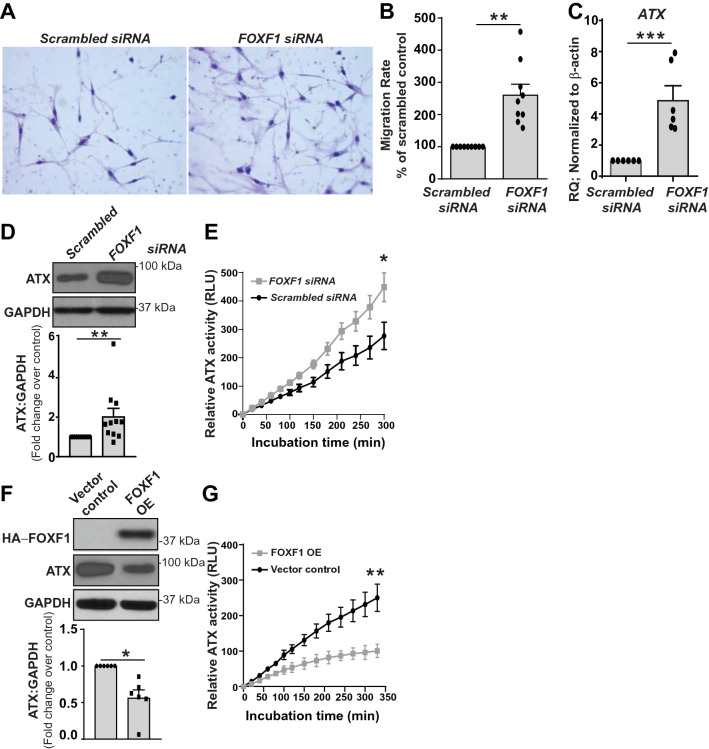
*FOXF1* silencing increases migration rates, and the expression and activity of ATX in LR-MSCs. (**A**) LR-MSCs invasion was analyzed using matrigel-coated transwells with 8 μm pore size. (**B**) Quantification of data in (**A**). Values: Means ± SEM; n = 9; **p < 0.0045. (**C**,**D**) *FOXF1* silencing upregulated the expression of ATX mRNA ((**C**); n = 6; ***p < 0.0003) and protein ((**D**); n = 11; **p < 0.0337) expressions. (**E**) The activity of ATX in the cell supernatant was assayed using the fluorogenic phospholipid substrate, FS-3. RFU: relative fluorescent unit, Values: Means ± SEM. n = 9, *p < 0.05. (**F**,**G**) DNA from pShuttle A-pCMV-HA-*mFoxf1* was utilized to overexpress *mFoxf1* in LR-MSCs, and the backbone vector pShuttle A was used as control. Immunoblotting analyses was utilized to confirm overexpression of FOXF1, and regulation of ATX and GAPDH ((**F**); n = 7; *p < 0.01). (**G**) ATX activity in cell supernatant is shown (n = 5; **p < 0.01). Values: Means ± SEM. Note: Full length blots for Fig. 2D and Fig. 2F are provided in Supplementary Fig. S3 and S4.

In order to ascertain the role of *FOXF1* in cellular migration, a functional in vitro assay using a modified Boyden chamber was utilized. Comparision of LR-MSCs transfected with scrambled control or *FOXF1-*specific siRNA demonstrated an approximate 2.5-fold increase in cell migration following *FOXF1-*silencing, suggesting that the loss of *FOXF1* imparts LR-MSCs with a robust migratory phenotype (Fig. 2A,B). ATX-LPA axis is key regulator of cellular migration and Autotaxin-encoding gene—*ENPP2* was noted to be among the top ten upregulated genes in *FOXF1*-silenced cells (Fig. 1I). Increased ATX expression in response to *FOXF1*-silencing was confirmed at mRNA and protein level by real-time PCR and western blotting respectively (Fig. 2C,D). Furthermore, supernatant from *FOXF1*-silenced LR-MSCs demonstrated significantly higher ATX activity utilizing a fluorimetric substrate, FS-3, compared to scrambled siRNA controls (Fig. 2E). We next overexpressed *mFoxf1* in LR-MSCs and assessed ATX protein levels and activity by immunoblotting and fluorimetry, respectively. Efficacy of *mFoxf1* overexpression was confirmed by immunoblotting for HA as shown in Fig. 2F. A 40% decrease in ATX protein expression was noted in LR-MSCs overexpressing *mFoxf1* (Fig. 2F), with a concordant decrease in ATX activity compared to control vector (Fig. 2G). Together these findings demonstrated that decreased FOXF1 leads to increased LR-MSC migration and ATX secretion and activity.

### ATX/LPA/LPA1 signaling axis mediates increased migration rates in *FOXF1*-silenced LR-MSCs

**Figure 3:**
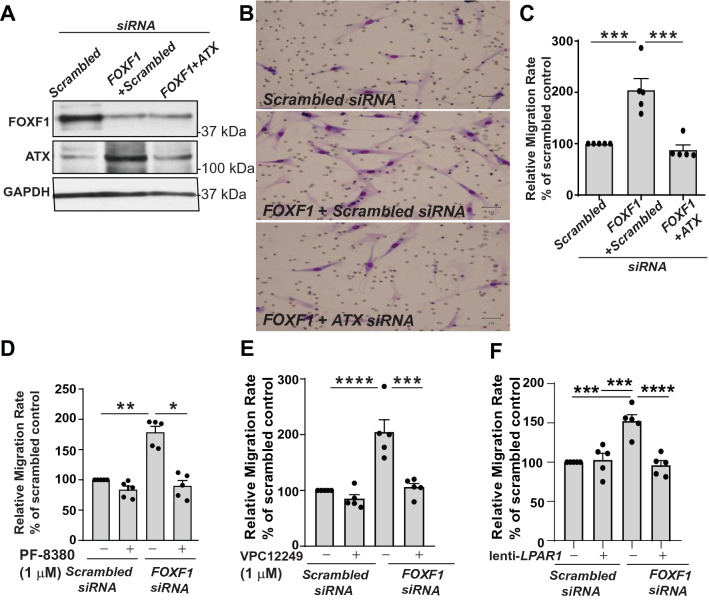
ATX-dependent cell migration in *FOXF1*-silenced LR-MSCs. (**A**) LR-MSCs were transfected with scrambled or *FOXF1*-specific siRNA. 24 h later, these LR-MSCs were transfected with scrambled or *ATX*-specific siRNA. Immunoblotting was performed to confirm *RNAi*-mediated *FOXF1* and *ATX* silencing efficacy. n = 5 per group. (**B**) Migration assay was conducted in LR-MSCs transfected with scrambled or siRNA specific to *FOXF1*, ATX, or both *FOXF1*- and *ATX*-specific siRNA. Representative images of cell migration are shown. (**C**) Quantification of (B), n = 5, ***p < 0.0003. (**D**) LR-MSCs transfected with scrambled or *FOXF1*-specific siRNA were treated with the ATX inhibitor, PF-8380 (1 μM) and migration assay was performed. Values: Means ± SEM. n = 5, **p < 0.0142, *p < 0.0325. (**E**,**F**) Migration assays are shown with LR-MSCs transfected with scrambled or *FOXF1*-specific siRNA, and then treated with the LPA1 inihibitor, VPC12249 (1 μM) (**E**), or subjected to lentivirus-mediated shRNA interference against *LPAR1* (**F**). Values: Means ± SEM. n = 5. ***p < 0.0002, ****p < 0.0001. Note: Full length blots for Fig. 3A are provided in Supplementary Fig. S5.

To further ascertain if increased ATX expression mediates the increase in migration induced by loss of FOXF1, LR-MSCs transfected with *FOXF1* siRNA were subjected to subsequent transfection with siRNA specific to *ATX* or scrambled control (Fig. 3A). Migration was compared between LR-MSCs silenced for *FOXF1* alone or in combination with *ATX* silencing in the modified Boyden chamber migration assay (Fig. 3B). Increased migration noted in response to *FOXF1*-silencing was abrogated in LR-MSCs subjected to gene silencing for both *FOXF1* and *ENPP2 (ATX)* (Fig. 3C). That ATX is a key factor in mediating the pro-migratory effect of FOXF1 inhibition was further confirmed by using PF8380, a specific pharmacologic inhibitor of ATX. *FOXF1*-silenced LR-MSCs treated with PF8380 demonstrated significant reduction in migration rates with levels comparable to scrambled control siRNA transfected LR-MSCs (Fig. 3D).

ATX regulates cellular migration via its generation of lipid mediator LPA and downstream LPA receptor signaling. We have previously shown that LR-MSCs predominantly express LPA receptor isoform 1 (LPA1) and that migration of LR-MSCs in response to LPA is mediated via LPA1 receptor ligation^4^. To study the pharmacologic blockade of LPA signaling on migration of *FOXF1*-silenced LR-MSCs, we utilized VPC12249, an LPA1-specific antagonist. *FOXF1*-silenced LR-MSCs demonstrated a two-fold higher migration, which was significantly diminished by the pharmacologic blockade of LPA1 (Fig. 3E). Furthermore, lentiviral gene silencing of the LPA receptor—*LPAR1,* using short hairpin RNA (shRNA), was adopted as a complementary approach to determine the effects of LPA signaling on migration. *FOXF1-*silenced LR-MSCs demonstrated 1.5-fold higher migration, which was significantly mitigated by shRNA-mediated lentiviral repression of *LPAR1* gene expression (Fig. 3F)*.* Together, these data demonstrate that loss of FOXF1 promotes LR-MSC migration via ATX/LPA/LPA1 signaling pathway.

### Identification of novel *FOXF1* binding sites in the − 1.5 kb upstream region of the *ATX* promoter (− 1217/− 1127/− 458)

**Figure 4:**
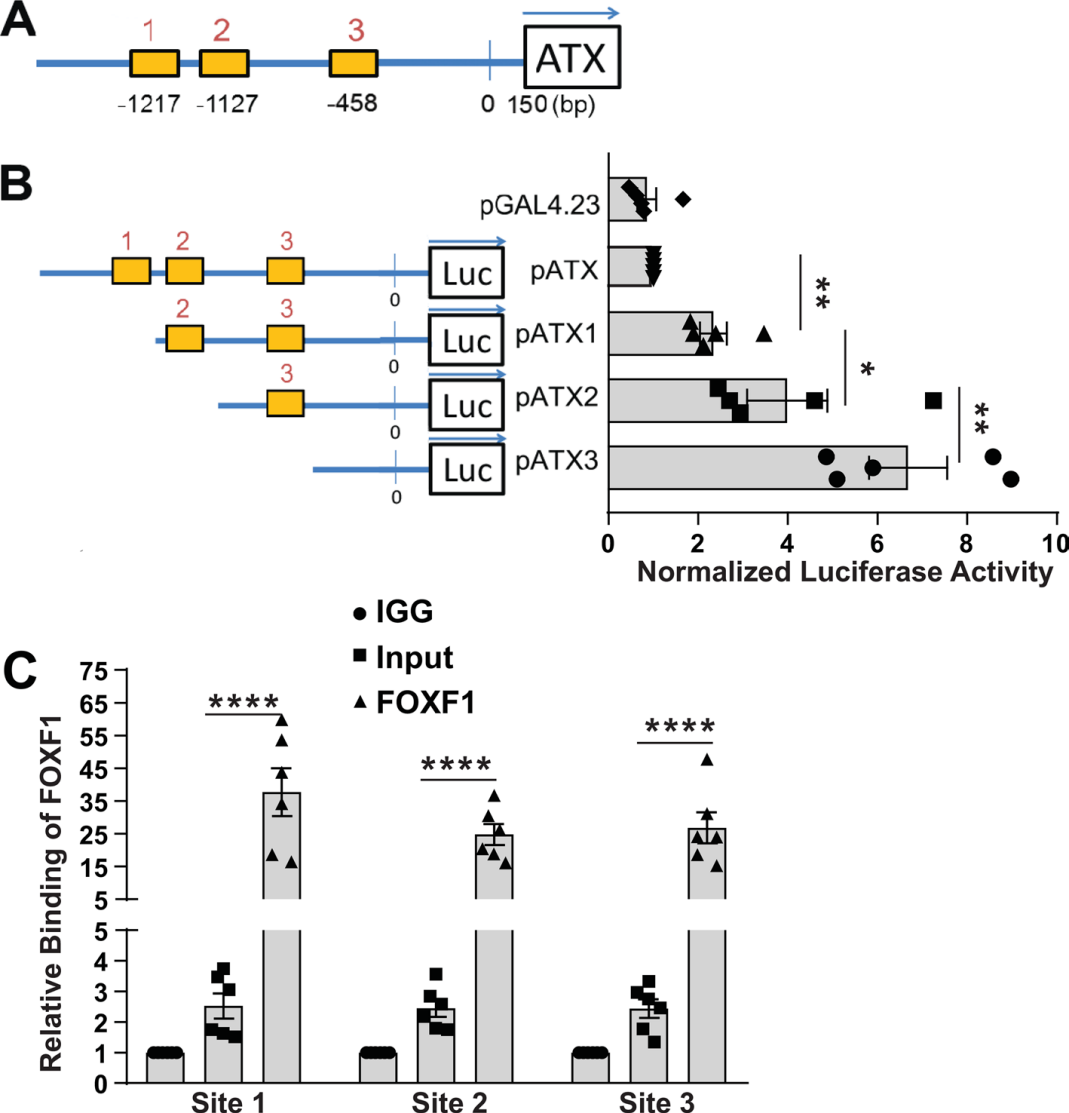
FOXF1 inhibits *ATX* transcription and directly binds to ATX promoter. (**A**) Three potential FOXF1 binding sites exist in the 1.5 kb ATX promoter and their locations upstream the ATX transcription initiation site were marked. (**B**) Three versions of truncated ATX promoter were utilized to drive luciferase expression in LR-MSCs and corresponding luciferase activities were measured, n = 5, *p < 0.05, **p < 0.01. (**C**) ChIP assay was performed to detect the direct binding of FOXF1 with its three potential binding sites in the ATX promoter. Values: Means ± SEM. ****p < 0.0001.

Silencing of *FOXF1* in LR-MSCs resulted in a robust induction of ATX at mRNA level, so we next sought to investigate whether *FOXF1* directly binds to regions of the *ATX* promoter to influence transcription. Utilizing JASPAR promoter analysis (www.jaspar.genereg.net), we observed three putative *FOXF1/2* binding sites (RTAAAYA)^21^ in the − 1.5 kb upstream region of *ATX* promoter (Fig. 4A). To study the function of these three potential binding sites, we constructed *luc2* luciferase expressing vectors driven by full length or truncated *ATX* promoters on the pGAL4.23 backbone (Fig. 4B). These reporter constructs were co-transfected with control Renilla vector pRL-TK into LR-MSCs and the luciferase activity was measured. As shown in Fig. 4B, deletion of the two most upstream putative *FOXF1* binding sites (sites 1 and 2) increased luciferase expression two and fourfold, respectively. Promoter truncation omitting all 3 putative FOXF1 binding sites resulted in a sixfold induction of *ATX* transcription, suggesting a role for all three binding sites in repressing *ATX* transcription. Next, we utilized chromatin immunoprecipitation (ChIP) analysis to investigate if *FOXF1* can bind these putative sites in the *ATX* promoter. FOXF1 antibody was used to pull down the FOXF1/chromosome complexes with goat IgG as the negative control. ChIP data demonstrated an over 20-fold increase in FOXF1 binding at all three sites of the *ATX* promoter compared to IgG control (Fig. 4C). Collectively, these results suggest that FOXF1 transcriptionally represses expression of *ATX* by directly binding to regions of the *ATX* promoter.

### *FOXF1*-silencing induces proliferation and inflammatory responses in LR-MSCs

**Figure 5:**
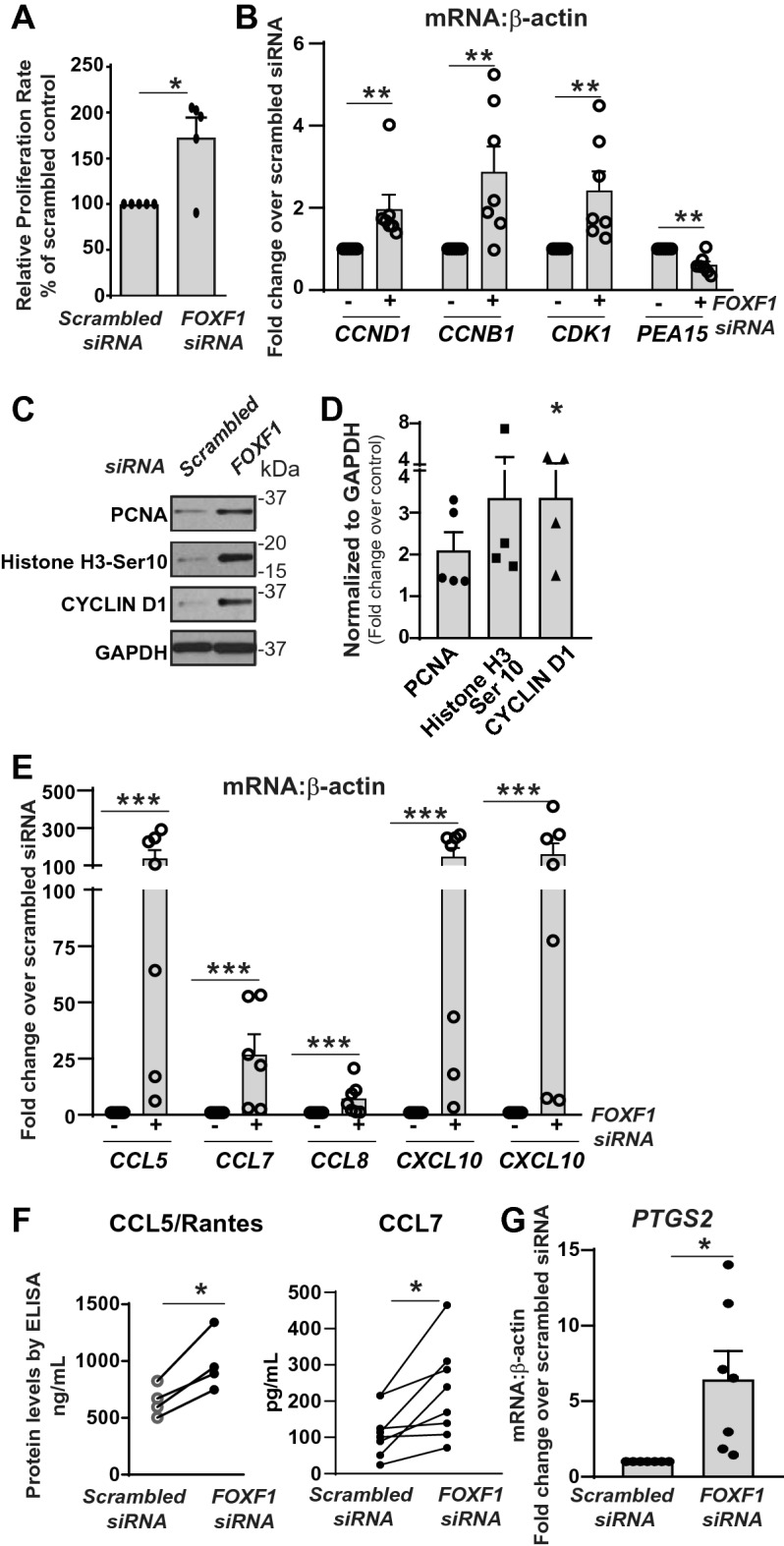
*FOXF1* silencing promotes cellular proliferation and secretion of inflammatory mediators in LR-MSCs. (**A**) Proliferation rate was analyzed using the CyQUANT NF Cell Proliferation Assay. n = 5; values: Means ± SEM. *p < 0.05. (**B**) Real-time PCR analyses of specific genes detected in the Affymetrix array analysis. Values: Means ± SEM; n = 7; **p < 0.01. (**C**) Protein lysates with equal concentrations (~ 10 µg) from LR-MSCs transfected with *FOXF1-*specific or scrambled siRNA were subjected to immunoblotting against proliferation markers—anti-PCNA, anti- phosphorylated histone H3 (Ser 10), anti-cyclin D1 and anti-GAPDH (loading control). (**D**) Quantification of data in (**C**) is shown as fold change over scrambled control. Values: Means ± SEM; n = 4; *p < 0.05. (**E**) LR-MSCs were transfected with scrambled or *FOXF1* siRNA and subjected to real-time PCR analyses of key cytokines. Values: Means ± SEM. n = 7, ***p < 0.001. (**F**) Secreted CCL5 and CCL7, in the conditioned media from (**E**) were measured by ELISA. Values: Means ± SEM. n = 4 (CCL5) and n = 8 (CCL7). *p < 0.05. (**G**) Real-time PCR analyses of *PTGS2*. Values: Means ± SEM. n = 7, **p < 0.01. Note: Full length blots for Fig. 5C are provided in Supplementary Fig. S6.

We also further investigated other biological functions which were identified to be significantly altered by *FOXF1* silencing by affymetrix analyses (Table 2). Quantitative assessment of cellular proliferation by CyQUANT NF cell proliferation assay demonstrated approximately 75% higher proliferation in *FOXF1-*silenced LR-MSCs compared to that of the scrambled siRNA control (Fig. 5A). Real-time PCR analyses confirmed upregulation of genes involved in cell cycle progression upon *FOXF1* silencing, such as *cyclin D1 (CCND1)*, *cyclin B1 (CCNB1)*, *cyclin-dependent kinase 1 (CDK1)* and *phosphoprotein enriched in astrocytes 15 (PEA15*) (Fig. 5B). Additionally, *FOXF1-*silencing in LR-MSCs demonstrated upregulation of proteins marking proliferation and cell cycle progression such as proliferating cell nuclear antigen (PCNA), phosphorylated histone H3 (Ser 10) and cyclin D1 (Fig. 5C,D).

The gene expression pattern demonstrating increased pro-inflammatory cytokines noted in *FOXF1-*silenced LR-MSCs by Affymetrix analyses was also further confirmed by real-time PCR**.** An approximately 100-fold increase in the gene expression of *CCL5* and tenfold increase in the gene expression of *CCL7*, and a 150-fold increase in the gene expressions of CXCL10 and CXCL11 was found in *FOXF1*-silenced LR-MSCs relative to scrambled siRNA control (Fig. 5E). *FOXF1*-silencing induced increase in cytokine secretion by LR-MSCs was documented by ELISA where higher levels of CCL5 and CCL7 were noted in conditioned media collected from *FOXF1-*silenced LR-MSCs compared to the respective scrambled controls (Fig. 5F). Real-time PCR also confirmed that loss of FOXF1 induced expression of *Prostaglandin-Endoperoxide Synthase 2* (*PTGS2 or COX2) a* key enzyme in prostaglandin biosynthesis (Fig. 5G).

## Discussion

Mesenchymal cells are a critical component of cellular niches in all organs and play a key role in the pathogenesis of fibrotic diseases^22^. However, transcriptional networks and signaling mechanisms involved in regulating mesenchymal progenitor cells in homeostatic conditions are not well identified. Here, we identify a role for transcription factor forkhead protein FOXF1 as a master repressor of key cellular functions in human LR-MSCs. *FOXF1* silencing was noted to promote proliferation, migration, and secretory function of LR-MSCs. Furthermore, FOXF1 was identified as a novel transcriptional repressor of ATX, a key enzyme largely responsible for the synthesis of extracellular pro-fibrotic mediator, LPA. Increased ATX secretion followed by subsequent LPA synthesis and autocrine LPA1 signaling, mediated LR-MSC migration in response to decreased FOXF1 expression. Together, these data shed light on novel restraining mechanisms in mesenchymal cells which limit their activation in homeostatic conditions. These findings have significant relevance to understanding both adaptive and mal-adaptive reparative processes in the lung.

Our studies provide first evidence for the role of FOXF1 as a transcriptional repressor of key enzyme ATX in human LR-MSCs. ATX, a secreted glycoprotein from the family of ectonucleotide pyrophosphatases/phosphodiesterases, is essential for development and is implicated in a variety of physiologic and pathologic processes^23^. ATX produces majority of the extracellular LPA and the ATX/LPA/LPA1 signaling axis has been shown to play a key role in fibrosis, inflammation, and cancer across various organs^5,24–31^. ATX-LPA signaling is implicated in fibrotic diseases of the lung^5,30,32^, and we have demonstrated stable increased expression of ATX in mesenchymal cells derived from fibrotic lung allografts^5^. In these studies, *ATX* mRNA expression was noted to be regulated by nuclear factor of activated T cells 2 (NFAT1). NFAT1 is a known enhancer of ATX transcription with NFAT binding sites described in the *ATX* promoter region in breast cancer cells^33^. Other transcription factors such as HOXA13, v-JUN, NF-κB and Stat3 have also been identified as transcriptional activators of ATX in various murine and human cellular conditions^33–36^, however, no ATX repressor has been reported to date. ATX as a target of FOXF1 was identified by global affymetrix analysis where *ENPP2* was among the top differentially expressed genes in *FOXF1-*silenced LR-MSCs. We utilized both FOXF1 silencing and overexpression strategies to confirm regulation of ATX by FOXF1 in LR-MSCs. Silencing of *FOXF1* resulted in robust increases in ATX at the transcriptional level as well as increased ATX expression and function—as indicated by increased ATX mRNA, protein, and activity. FOXF1 overexpression was associated with reduced ATX expression at both the RNA and protein level. We identified, previously uncharacterized, three putative *FOXF1* binding sites on the *ATX* promoter. That FOXF1 binds to and is a repressor of the ATX gene ENPP2 was confirmed by its direct binding to the ATX promoter using ChIP analysis. Increases in *ATX* transcription was noted in luciferase assays with subsequent promoter truncations. Future studies will focus on identifying the exact binding site. Previous studies in NIH3T3 cells have identified FOXF1 as a repressor of the CDH11 gene^37^ and other members of the FOX family such as FOXP1 and FOXP2 which are expressed in the lung epithelium have also been characterized as transcriptional repressors^38^.

A key finding of our work is recognition of the role of transcriptional factor FOXF1 as a inhibitory regulator of LR-MSC migration. Downregulation of FOXF1 resulted in a robust migratory phenotype in LR-MSCs which was found to be dependent on ATX secretion and downstream LPA/LPA1 signaling. Mesenchymal cell migration is a key feature of its activated state and its positive regulation by growth factors and biological mediators is well studied in context of tissue repair and fibrosis. However, the fundamental question of what prevents activation of mesenchymal cell migration in a quiescent condition has not been previously explored. Our data demonstrating FOXF1 as a transcriptional repressor of ATX and its loss promoting ATX/LPA/LPA1 signaling axis mediated migration suggests that FOXF1 expression could be critical brake on cellular migration in homeostatic conditions by keeping autocrine ATX expression in check. Loss of FOXF1 has been linked to increased invasiness of hepatocellular cancer cells^39^. FOXF1 has also been identified as a target of p53 in a separate study of human cancer cell lines, with its ectopic expression inhibiting cancer cell invasion and migration and its inactivation of FOXF1 stimulating cell invasion and migration^40^.

MSCs are key components of cellular niches, and regulate biologic processes via their paracrine actions and locally generated ATX has been demonstrated to be important in cellular interactions within tissue microenvironment^41^. Further evidence for the role of FOXF1 in regulating the secretome of the LR-MSCs was provided by affymetrix analysis where a significant change in the cytokine transcriptome was noted with marked upregulation of key chemokines such as CCL5, CCL7, CXCL10 and CXCL11. PTGS2, the enzyme that regulates prostanoid synthesis, was also significantly upregulated in *FOXF1*-silenced LR-MSCs. This suggests that FOXF1 regulates multiple downstream pathways in human LR-MSCs, the mechanism of which remains to be elucidated. Future studies are needed to identify other transcriptional targets of FOXF1 in LR-MSCs.

Among top upregulated biological processes identified in *FOXF1-*silenced LR-MSCs by GO analysis were positive regulation of cell proliferation. Mesenchymal cells within the lungs have relatively low turnover^42^, but our previous longitudinal studies of human lung allografts have provided clues regarding conditions associated with LR-MSC proliferation and mobilization^3^. An increase in LR-MSC numbers were noted early post-transplant during an active repair phase and later post-transplant preceding development of allograft fibrosis^3^. Both these conditions are marked by significant epithelial injury and FOXF1 plays a key role in mesenchymal-epithelial interactions during lung development^43^. FOXF1 is a Shh target gene and loss of Shh signaling has been implicated in mesenchymal cell proliferation in murine models^44,45^. Our finding that loss of FOXF1 promotes cellular proliferation suggests that FOXF1 could be a key intermediary for the actions of Shh. That loss of FOXF1 can promote mesenchymal cell activation and contribute to fibrosis is suggested in studies of transgenic mice with myofibroblast-specific deletion of *Foxf1,* where worse fibrotic remodeling was noted in response to bleomycin^37^. Further investigations are needed to shed more light on the regulation of this novel regulatory mechanism of mesenchymal cell activation in normal reparative and aberrant fibrotic responses within tissue niches in a human lung.

In conclusion, our study elucidates a critical mechanistic role of transcription factor *FOXF1* that acts as a master regulator of cellular functions and paracrine actions of resident MSCs in human adult lungs. Furthermore, these studies are novel in their elucidation of the first transcriptional repressor of *ATX* in any cell type, a finding that has significant implication across various organs and diseases.

## Supplementary information

Supplementary Figures.

## Data Availability

The data that support the findings of this study are available from the corresponding author upon reasonable request.

## Abbreviations

FOXF1: Forkhead box isoform 1
ATX: Autotaxin
*ENPP2*: Ectonucleotide pyrophosphatase/phosphodiesterase 2 (encodes for ATX)
LPA: Lysophosphatidic acid
LPA1: LPA receptor isoform 1
LR-MSCs: Lung-resident mesenchymal cells
*mFoxf1*: Murine FOXF1 overexpressing vector
MOI: Multiplicities of infection
DMEM: Dulbecco’s minimum essential medium
FBS: Fetal bovine serum
PCNA: Proliferating cell nuclear antigen
HRP: Horseradish peroxidase
ChIP: Chromatin immunoprecipitation
CCL5/RANTES: Chemokine ligand 5
CCL7/MCP-3: Chemokine ligand 7
STRING: Search tool for the retrieval of interacting genes/proteins
GO: Gene ontology
CCND1: Cyclin D1
CCNB1: Cyclin B1
CDK1: Cyclin dependent kinase 1
PEA15: Phosphoprotein enriched in astrocytes 15
PTGS2: Prostaglandin-endoperoxide synthase 2
COX2: Cyclooxygenase isoform 2
PGE2: Prostaglandin E2
NFAT1: Nuclear factor of activated T cells 2
HOXA13: Homeobox A13
CDH11: Cadherin 11 or osteoblast-cadherin
CXCL10: C-X-C Motif chemokine ligand 10
CXCL11: C-X-C Motif chemokine ligand 11
