# Supplementary material for: Loss of FOXF1 expression promotes human lung-resident mesenchymal stromal cell migration via ATX/LPA/LPA1 signaling axis
Source: Sci Rep. 2020 Dec 4;10:21231. doi: 10.1038/s41598-020-77601-1 (PMC7718269; doi:10.1038/s41598-020-77601-1)
Supplement: Supplementary file 1 — Supplementary Figures. [file 41598_2020_77601_MOESM1_ESM.pdf]

Loss of FOXF1 expression promotes human lung-resident mesenchymal stromal cell migration via ATX/LPA/LPA1 signaling axis

Pengxiu Cao, Natalie M. Walker, Russell R. Braeuer, Serina Mazzoni-Putman, Yoshiro Aoki, Keizo Misumi, David S. Wheeler, Ragini Vittal, and Vibha N. Lama

Division of Pulmonary and Critical Care Medicine, Department of Internal Medicine, University of Michigan Health System, Ann Arbor, MI, USA

SUPPLEMENTARY MATERIAL

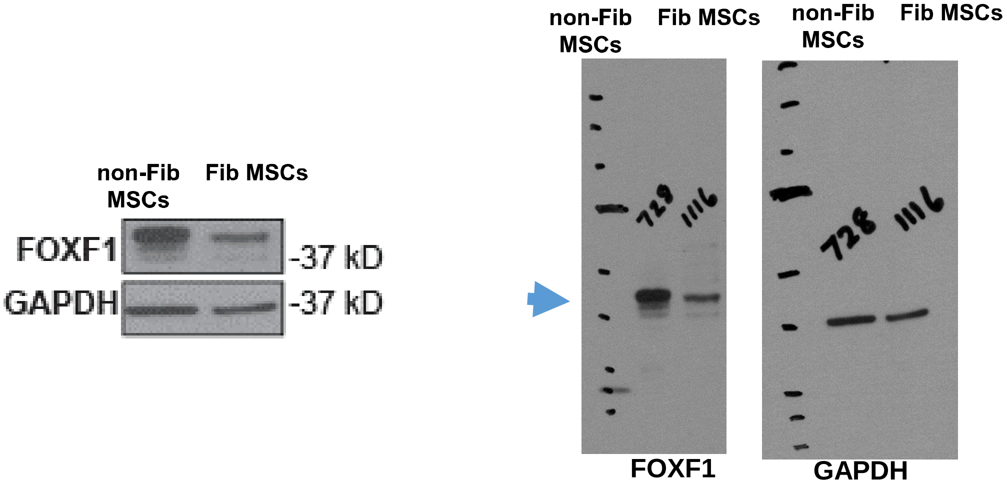

Supplementary Figure S1. Full length blot for Figure 1B

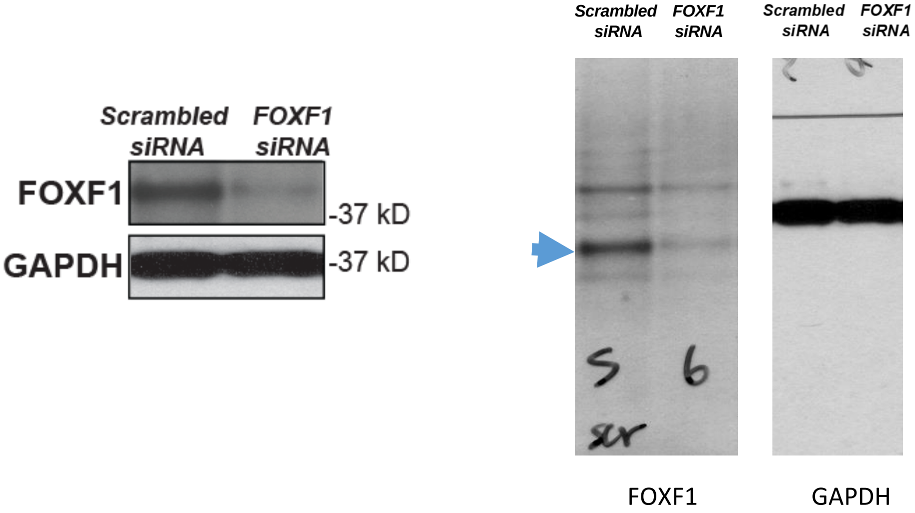

Supplementary Figure S2. Full length blot for Figure 1D

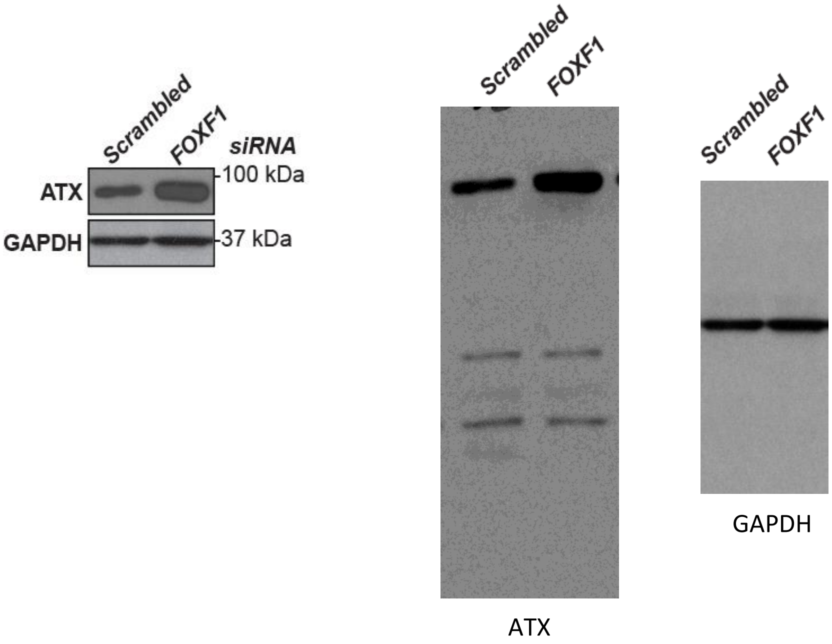

Supplementary Figure S3. Full length blot for Figure 2D

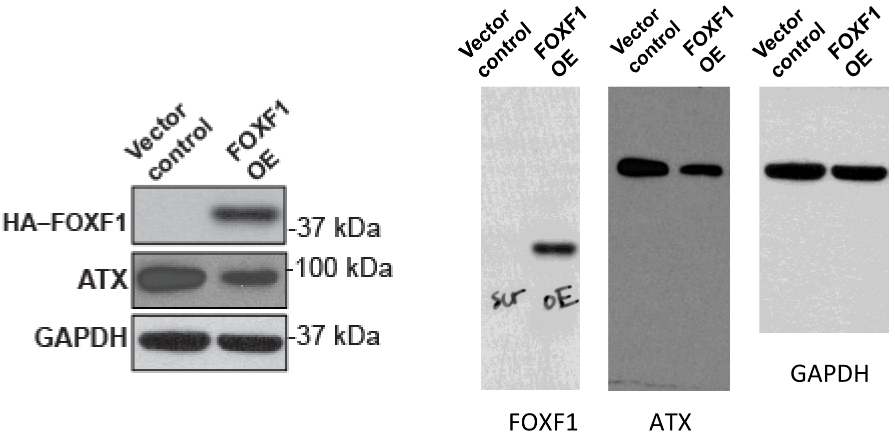

Supplementary Figure S4. Full length blot for Figure 2F

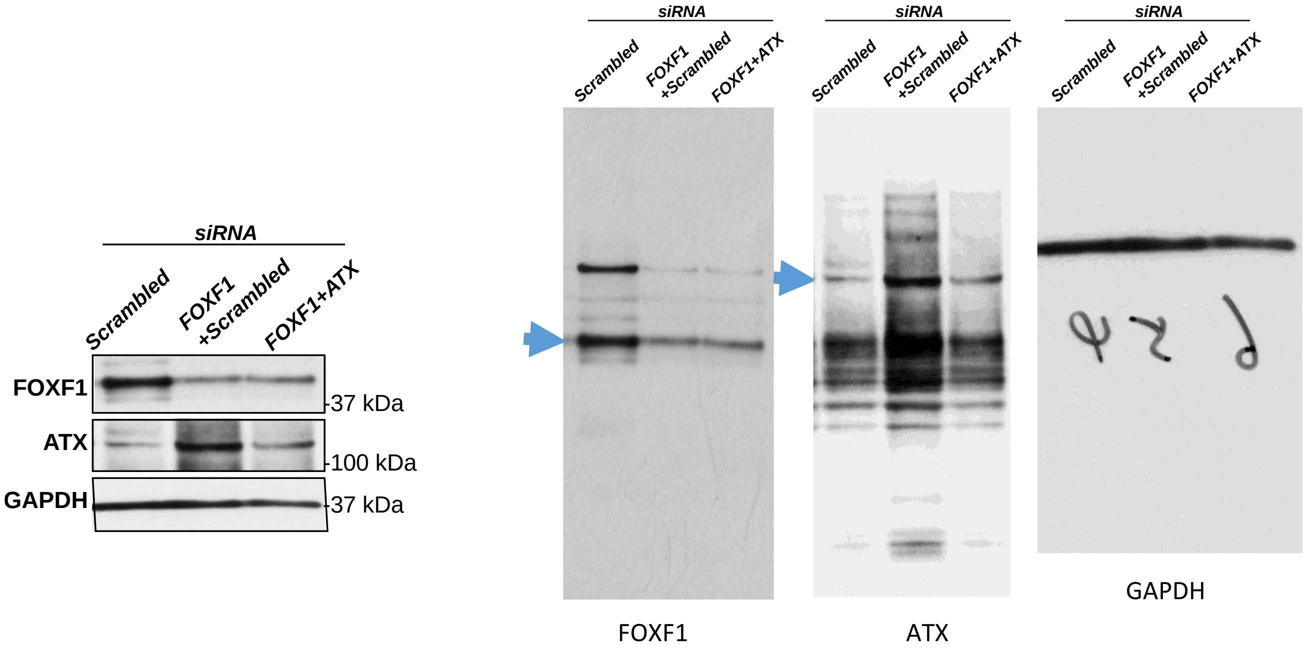

Supplementary Figure S5. Full length blot for Figure 3A

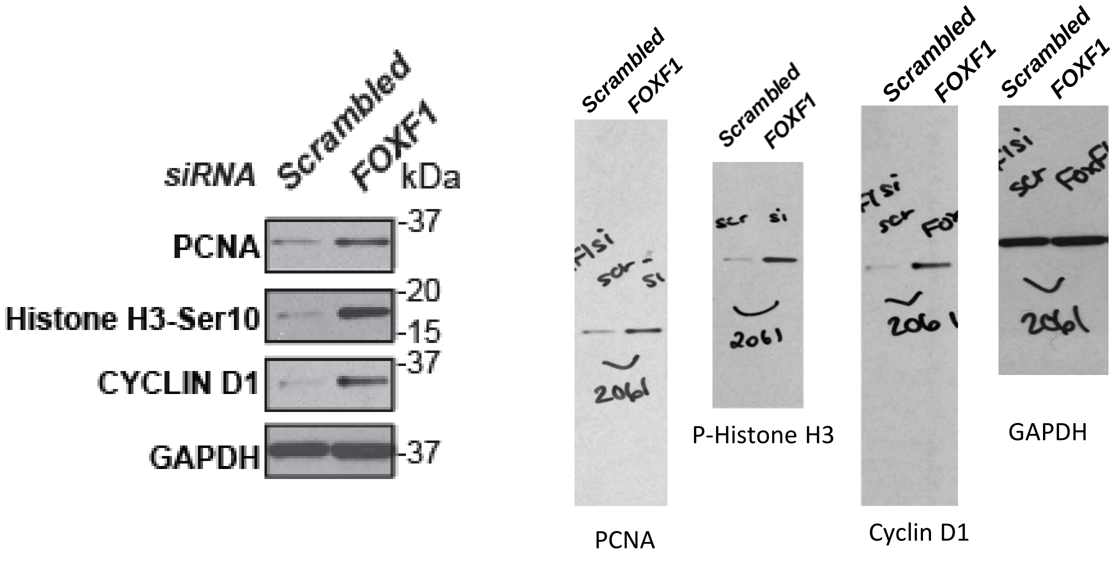

Supplementary Figure S6. Full length blot for Figure 5C
